# Dasatinib, a Src inhibitor, sensitizes liver metastatic colorectal carcinoma to oxaliplatin in tumors with high levels of phospho-Src

**DOI:** 10.18632/oncotarget.8880

**Published:** 2016-04-20

**Authors:** Marco Perez, Antonio Lucena-Cacace, Luis Miguel Marín-Gómez, Javier Padillo-Ruiz, Maria Jose Robles-Frias, Carmen Saez, Rocio Garcia-Carbonero, Amancio Carnero

**Affiliations:** ^1^ Instituto de Biomedicina de Sevilla, IBIS/Hospital Universitario Virgen del Rocío/ Universidad de Sevilla/Consejo Superior de Investigaciones Científicas, Seville, Spain; ^2^ Department of General Surgery, Virgen del Rocío University Hospital, Seville, Spain; ^3^ Department of Pathology, Virgen del Rocío University Hospital, Seville, Spain; ^4^ Present address: HUVR-IBiS Biobank, Virgen del Rocío University Hospital, Seville, Spain; ^5^ Department of Medical Oncology, Virgen del Rocío University Hospital, Seville, Spain; ^6^ Present address: Department of Medical Oncology, 12 of October University Hospital, Madrid, Spain

**Keywords:** metastatic colorectal carcinoma, cancer treatment, biomarkers, Src kinase, pdx models

## Abstract

Despite the development of new antineoplastic agents for the treatment of colorectal cancer (CRC), oxaliplatin and fluoropyrimidines remain the most commonly employed drugs for the treatment of both early and advanced disease. Intrinsic or acquired resistance is, however, an important limitation to pharmacological therapy, and the development of chemosensitization strategies constitute a major goal with important clinical implications. In the present work, we determined that high levels of activated Src kinase, measured as phospho-Src at the Tyr419 residue in CRC cell lines, can promote colorectal carcinoma cell resistance to oxaliplatin, but not to 5-fluorouracil (5FU), and that inhibition of this protein restores sensitivity to oxaliplatin. Similar results were observed with *in vivo* patient-derived xenograft (PDX) models that were orthotopically grown in murine livers. In PDX tumor lines derived from human CRC liver metastasis, dasatinib, a Src inhibitor, increases sensitivity to oxaliplatin only in tumors with high p-Src. However, dasatinib did not modify sensitivity to 5FU in any of the models. Our data suggest that chemoresistance induced by p-Src is specific to oxaliplatin, and that p-Src levels can be used to identify patients who may benefit from this combination therapy. These results are relevant for clinicians as they identify a novel biomarker of drug resistance that is suitable to pharmacological manipulation.

## INTRODUCTION

Metastatic CRC (mCRC) remains incurable for patients with surgically unresectable disease. Several combination regimens including fluoropyrimidines, oxaliplatin and/or irinotecan, with or without monoclonal antibodies targeting vascular endotelial growth factor (VEGF) or epidermal growth factor receptor (EGFR), remain the mainstay of care in metastatic CRC (mCRC). Response rates, however, are observed in only 40–60% of the patients, and even responding patients inevitably develop refractory disease, with median overall survival that does not generally exceed 2-2,5 years.

Oxaliplatin, one of the most commonly used drugs in the treatment of CRC, is a platinum-based chemotherapeutic agent that forms platinum-DNA adducts that block DNA replication, leading to cell cycle arrest and cell death [1–4]. Resistance to platinum agents occurs through several mechanisms, including decreased platinum influx, improved base excision repair, and/or increased detoxification by glutathione and metallothionein [1, 3]. Reversing resistance has proved to be challenging, due in part to ineffective pharmacological modulation of these pathways. Recently, however, Src family kinases have been implicated in drug resistance [5, 6]. Src is the prototype of this nine-member family, and it is activated by numerous growth migratory, and stress pathways [7]. In CRC cell lines and primary tumors, both c-Src kinase activity and protein expression levels have been found to be elevated compared with either normal colonic mucosal cells and fibroblasts or normal adjacent tissue [8–10]. However, c-Src seems to experience a robust increase in expression and activity in CRC liver metastases compared with metastases derived from other tumors types [11, 12], and increased c-Src levels correlate with worse patient survival [13]. The Src proteins belong to a family of non-receptor cytoplasmic protein tyrosine kinases that act as signaling complexes, which recruit and participate in intracellular signal transduction pathways related to carcinogenesis, as well as cell proliferation and differentiation, angiogenesis, migration, invasion, adhesion and apoptosis [14–16]. Phosphorylation and dephosphorylation regulate Src activity. In its inactive form, the end carboxyl-terminal Tyr527 residue is phosphorylated by protein tyrosine kinase Csk. Dephosphorylation at this level induces a conformational change in the protein, allowing the autophosphorylation of Tyr at residue 419, which is present in the activation loop, promoting and activating Src kinase (p-Src) [17, 18]. In normal basal conditions, 90-95% of Src es found in its inactive conformation. However, mutations at residue Tyr527 and the SH2 and SH3 domains, or alterations in Csk kinase activity, lead to constitutively activated p-Src. Whereas Src has been implicated in a myriad of cellular processes that are deregulated in cancer, current evidence suggests that Src activation is critical to tumor progression and metastasis [16]. In CRC, Src deregulation primarily involves protein overexpression, although the molecular mechanisms have not been fully elucidated. In addition, evidence from preclinical studies in sarcoma and ovarian cancer cell lines suggests that activation of Src reduces sensitivity to various chemotherapeutic drugs, including platinum agents, and that this resistance can be reversed by pharmacological inhibition [19, 20][21–23][24]. For example, treatment with the Src inhibitor PP2 reversed cisplatin resistance in a multidrug-resistant ovarian carcinoma cell line compared with its isogenic control [25]. Moreover, expression of a dominant negative, kinase-defective Src mutant resulted in increased sensitivity to oxaliplatin-mediated apoptosis in KM12L4 human colon tumor cells *in vitro* [21]. As a result, coupled with the recent availability of relatively non-toxic Src family inhibitors, numerous clinical trials have been initiated to evaluate small molecule Src family inhibitors in solid tumors [24, 26].

The overall objective of our work is to evaluate the effect of Src inhibition *in vivo* and correlate effective Src inhibition with a biomarker that can be used in patients. With this purpose, we first explored the expression of p-Src in CRC cell lines and assessed its role as a predictor of resistance to currently used therapies. We also investigated the use of Src inhibitors as chemosensitizers to these therapies *in vitro* and in patient-derived xenografts, PDX, and compared the efficacy with p-Src activation in these tumors. Predicting the tumors in which Src inhibition may be a valuable addition to oxaliplatin-based chemotherapeutic regimens, and understanding the mechanisms by which this occurs shall provide clinicians useful tools to improve the identification of patients who may benefit from Src inhibitors.

## RESULTS

### Effect of oxaliplatin in a panel of CRC cell lines

**Figure 1:**
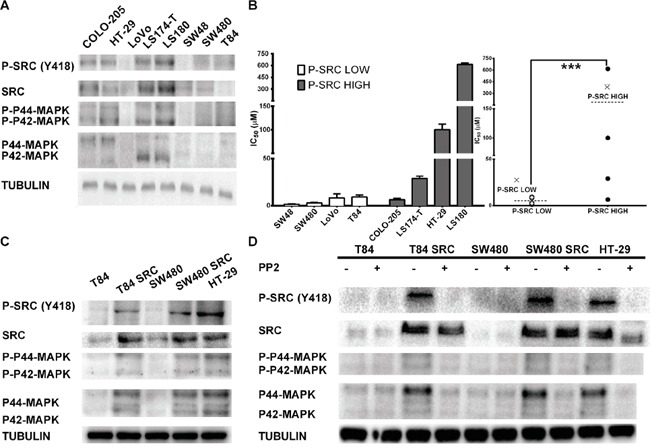
**A.** Characterization of the levels of phospho-Src in the CRC cell lines used in this study. Western blots were performed to measure p-Src, Src and p42/p44 MAPK protein levels. **B.** The cytotoxic effects of oxaliplatin directly correlate with activated Src protein levels. (a) IC50 values are separated in two differentiated groups by Src levels. (b) IC50 values without SD are grouped into a fall plot representation. **C.** Overexpression of c-Src in CRC cell lines induces activation of the pathway. Western blot analysis was performed to evaluate Src protein (anti-Src), the activated form of Src kinase (phosphorylated SrcY419), and p42/p44 Map kinase in CRC cell lines with normal endogenous expression of Src and overexpression of Src through stable transfection (T84 and SW480). **D.** Src inhibitor PP2 inhibits Src activity. Western blotting was performed to evaluate Src protein (anti-Src), the activated form of Src kinase (phosphorylated SrcY419), and p42/p44 Map kinase in CRC cell lines with normal endogenous expression of Src and overexpression of Src through stable transfection (T84 and SW480).

To explore the effect of Src activation, we evaluated a panel of 8 colorectal carcinoma cell lines (Table 1, Suplementary Table S1). We first characterized the levels of active Src (Src protein phosphorylated at Tyr419, p-Src) and correlated this activation with downstream pathway effectors. We observed that 4 cell lines carry activated Src, p-Src, COLO205, HT29, LS174T and LS180. The remaining cell lines, LOVO, SW48, SW480 and T84, do not contain activated Src. The high levels of p-Src correlated with increased levels of total Src protein and maintained high levels of activated p42MAPK (Figure 1A). We also observed that p42MAPK activation correlated with p-Src but did not correlate with mutant Ras or Raf in these cell lines (Figure 1A).

**Table 1:** Colorectal adenocarcinoma cell line chemosensitivity to oxaliplatin

Cell line	IC_50_ (μM)
	Oxaliplatin
**SW48**	1.47 ± 0.4
**SW480**	5.50 ± 1.6
**LoVo**	8.29 ± 4.4
**T84**	8.20 ± 1.4
**COLO-205**	6.43 ± 1.2
**LS174-T**	29 ± 2.4
**HT-29**	100 ± 2.1
**LS180**	616 ± 6.2

Next, we treated the 8 CRC cell lines with different concentrations of oxaliplatin and obtained an IC50 for each cell line. The response rankings from low concentrations to concentrations higher than 100 μM, as in HT29 and LS180 are shown in Table 1. The IC50 values of each cell line were influenced by level of activated Src (SrcY419), with higher levels observed in chemoresistant cell lines as shown in Figure 1B. Values were also influenced by the levels of phosphorylated p42MAPK. However, the number of cell lines explored was too small to draw definitive conclusions. COLO-205 does not show oxaliplatin resistance despite expressing high levels of p-Src.

### Src overexpression induces chemoresistance to oxaliplatin in CRC cell lines

Although we examined a limited number of cell lines, which may limit the relevance of our findings, we constitutively overexpressed the c-Src gene to study the functional relationship between p-Src levels and cellular responses to oxaliplatin [27]. We performed this evaluation in 2 different CRC cell lines with low levels of endogenous p-Src, T84 and SW480 (Figure 1C, Supplementary Figure S1). We observed that c-Src overexpression led to activation of p-Src in these cell lines, with concurrent activation of MAPKs (Figure 1C), correlating with the previous observation (Figure 1A). Furthermore, the pharmacological inhibition of p-Src with a PP2 inhibitor [28, 29] causes MAPK inhibition in these cell lines (Figure 1D). These data seem to confirm that the activation of MAPKs in CRC cell lines is more dependent on p-Src levels than on Ras or Raf mutations.

To gain insight into the effect of p-Src activation on cell line survival in the presence of oxaliplatin, we treated these p-Src transfected cells lines with oxaliplatin. We observed that cell lines expressing high p-Src had a two-fold higher IC50 compared with parental cells expressing an empty vector (Table 2). However, the IC50 values did not reach the levels of other resistant cell lines, such as HT29. On the other hand, treatment with PP2 alone is not sufficient to induce high toxicity in these cells independent of the levels of p-Src (Table 2). Finally, in response to the combination with sublethal doses of PP2, cells overexpressing p-Src were resensitized to oxaliplatin (Table 2).

**Table 2:** Activation of Src induces chemoresistance to Oxaliplatin

Transfectant	IC_50_ (μM)			
	Ras/Raf status	PP2	Oxaliplatin	Oxaliplatin + PP2*
**SW480**	**Mutated** Ras	18,75 ± 3,2	5.50 ± 1.6	1.23 ± 1.3
**SW480 SRC**	**Mutated** Ras	15,58 ± 4,7	10.44 ± 2.3	3.29 ± 1.0
**T84**	**Mutated** Ras	13,42 ± 2,8	8.20 ± 1.4	1.8 ± 2.2
**T84 SRC**	**Mutated** Ras	15,58 ± 4,7	14.75 ± 2.1	6.39 ± 1.7
**HT-29**	**Mutated** Raf	10,23 ± 4,5	100 ± 2.1	8.97 ± 1.1

* IC50 of oxaliplatin was calculated at constant concentration of 10 microM PP2.

Chemosensitization to Oxaliplatin was observed through the addition of Src inhibitor PP2.

Because CRC patients are also treated with 5FU [30], we explored whether p-Src is also relevant to the *in vitro* response to this drug. The same cell lines were treated with 5FU, and their IC50s were calculated. We did not find a clear correlation between responses to this drug and p-Src levels, even in cells overexpressing p-Src (Table 3). In fact, inhibition of p-Src by PP2 did not clearly sensitize the cells to 5FU treatment (Table 3).

**Table 3:** Colorectal adenocarcinoma cell line chemosensitivity to 5 Fluorouracil (5FU)

IC_50_ (μM)			
Transfectant	5-Fu	PP2	5-Fu + PP2
**SW48**	16,1 ± 3,2	14,78 ± 3,6	16,4 ± 4,4
**SW480**	21,5 ± 3,4	18,75 ± 3,2	17,3 ± 2,4
**SW480 SRC**	25,1 ± 2,1	15,58 ± 4,7	12 ± 4,1
**LoVo**	17 ± 2,3	14,21 ± 3,3	18,5 ± 3,6
**T84**	19,3 ± 4,6	13,42 ± 2,8	9,8 ± 4,3
**T84 SRC**	12,7 ± 4,1	13,78 ± 3,5	7,3 ± 3,7
**COLO-205**	8,9 ± 1,6	12,88 ± 3,4	9,2 ± 2,4
**LS174-T**	27,42 ± 3,6	18,61 ± 4,2	23,47 ± 4,8
**HT-29**	23,9 ± 4,5	10,23 ± 4,5	8,7 ± 2,3
**LS180**	3,69 ± 1,7	16,41 ± 5,7	4,5 ± 2,6

*IC50 of 5FU was calculated at constant concentration of 10 microM PP2.

Our data suggest, therefore, that the chemoresistance induced by p-Src is specific to oxaliplatin.

### *In vivo* Src inhibition of liver metastasis of colorectal tumors

**Figure 2:**
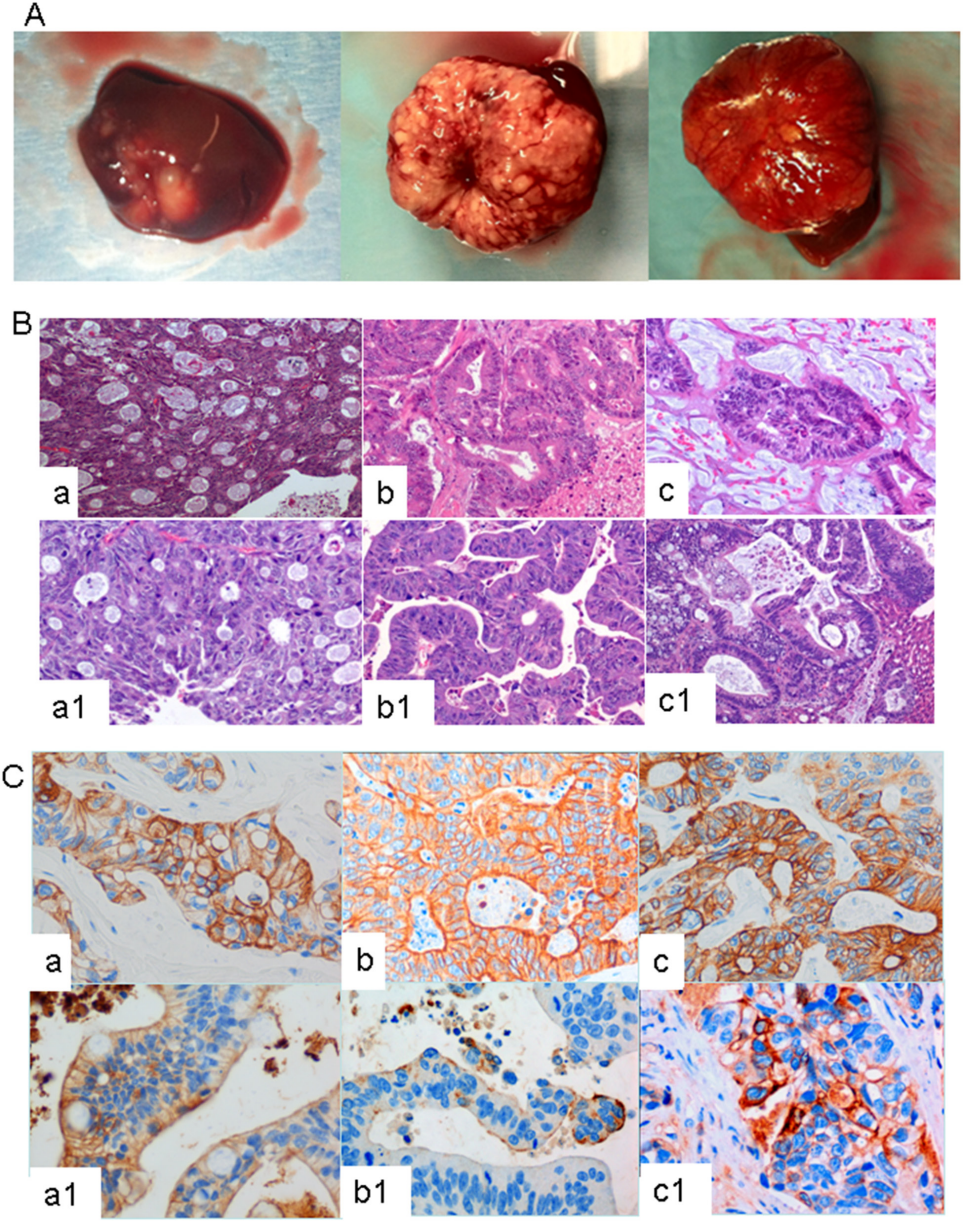
**A.** Three representative examples of CRC liver metastasis growing orthotopically in murine liver. **B.** and **C.** Human CRC liver metastasis and their derived PDX lines show similar pathological features. B) Hematoxylin and eosin staining and C) cytokeratin 20 staining of CRC human liver metastasis and their derived PDX lines. In these figures, a/b/c correspond to the human tumors, and a1/b1/c1, to the PDX derived tumors.

To explore the effect of Src inhibition *in vivo*, we used orthotopic PDX models that were generated from mCRC patients undergoing surgical resection of liver metastasis. Twenty-six tumor samples derived from these CRC liver metastases were surgically implanted into the livers of female nude mice (nu/nu) aged 4-6 weeks according to the protocol described in the Materials and Methods section. Of the 26 tumors implanted, 42% grew and were reimplanted according to a procedure that was identical to that used for primary sample placement. In all cases, we observed tumor growth in the liver (Figure 2A). PDXs grow orthotopically in the liver, maintaining the same architecture and histological features of the human metastasis from which they were derived (Figure 2B and 2C).

The molecular profile, including the analysis of Ras, Raf, PI3K or PDGFR mutations, and microsatellite instability (MSI), also showed identical behavior in the human and PDX models (data not shown).

**Figure 3:**
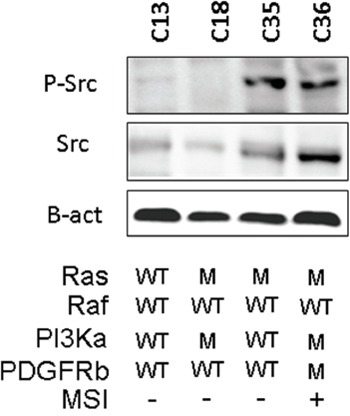
Relevant molecular characteristics of the PDX lines used in this study A western blot with the phospho-Src levels and the information about K-Ras, B-Raf, PI3Ka and PDGFRb mutation (M) or wild type (WT). The figure also shows whether (+) or not (−) the tumor present microsatellite instability, MSI.

From this panel, we selected 4 PDX lines (Figure 3), two with high p-Src and two with low p-Src expression. One model, C13, which had low p-Src expression, lacked mutations in Ras, Raf, PI3K, PDGFR, and had not MSI (Figure 3), whereas C18, which had also low p-Src expression, had mutations in Ras and PI3Ka and lacked mutations in Raf and MSI. On the other hand, both models with high p-Src expression, C35 and C36, had mutations in Ras and lacked mutations in Raf. C36 also showed activating mutations in PI3Ka.

**Figure 4:**
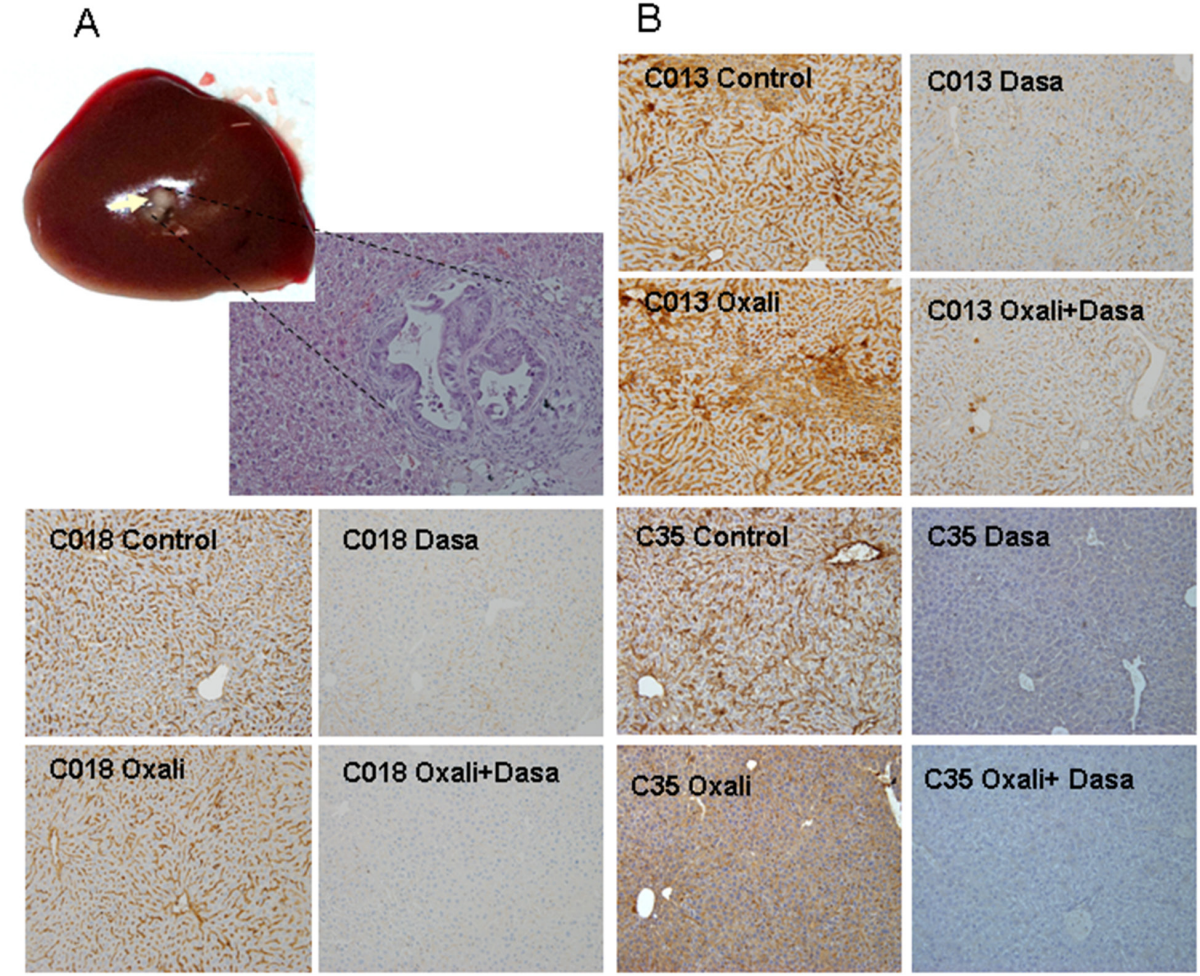
**A.** Orthotopic engraftment of human CRC liver metastasis in murine livers at the initiation of treatment. The four liver metastases of CRC tumor-derived PDXs were orthotopically engrafted into murine liver and grown for 15 days to ensure proper engraftment. One mouse from each cohort was sacrificed to examine tumor engraftment. Mice were sacrificed and tumors analyzed. Picture shows representative macroscopic tumor engraft growing in liver (arrow) and a micrograph of tumor tissue of the same sample. This was repeated in all cohorts to ensure equal engraftment. **B.** Decrease in the levels of activated p-Src in the livers of treated mice. After 4 weeks of treatment, one mice of each cohort was sacrificed and the necropsy done. The liver was fixed and analyzed by immunohistochemistry for efficacy of pSrc inhibition.

The four liver metastases of CRC tumor-derived PDXs were orthotopically engrafted into murine liver and grown for 15 days to ensure proper engraftment. One mouse from each cohort was sacrificed to examine tumor engraftment (Figure 4A). Then, the animals were treated with oxaliplatin, the p-Src inhibitor dasatinib, a combination of both drugs (oxaliplatin plus dasatinib) or solvent (untreated control). On the day after the final dose, one mice harboring each tumor subtype that were treated with solvent or any of the treatments were sacrificed, and their tissues were analyzed for the expression of p-Src in the liver. We found a clear effect of p-Src inhibition in the livers of mice treated with dasatinib (Figure 4B).

**Figure 5:**
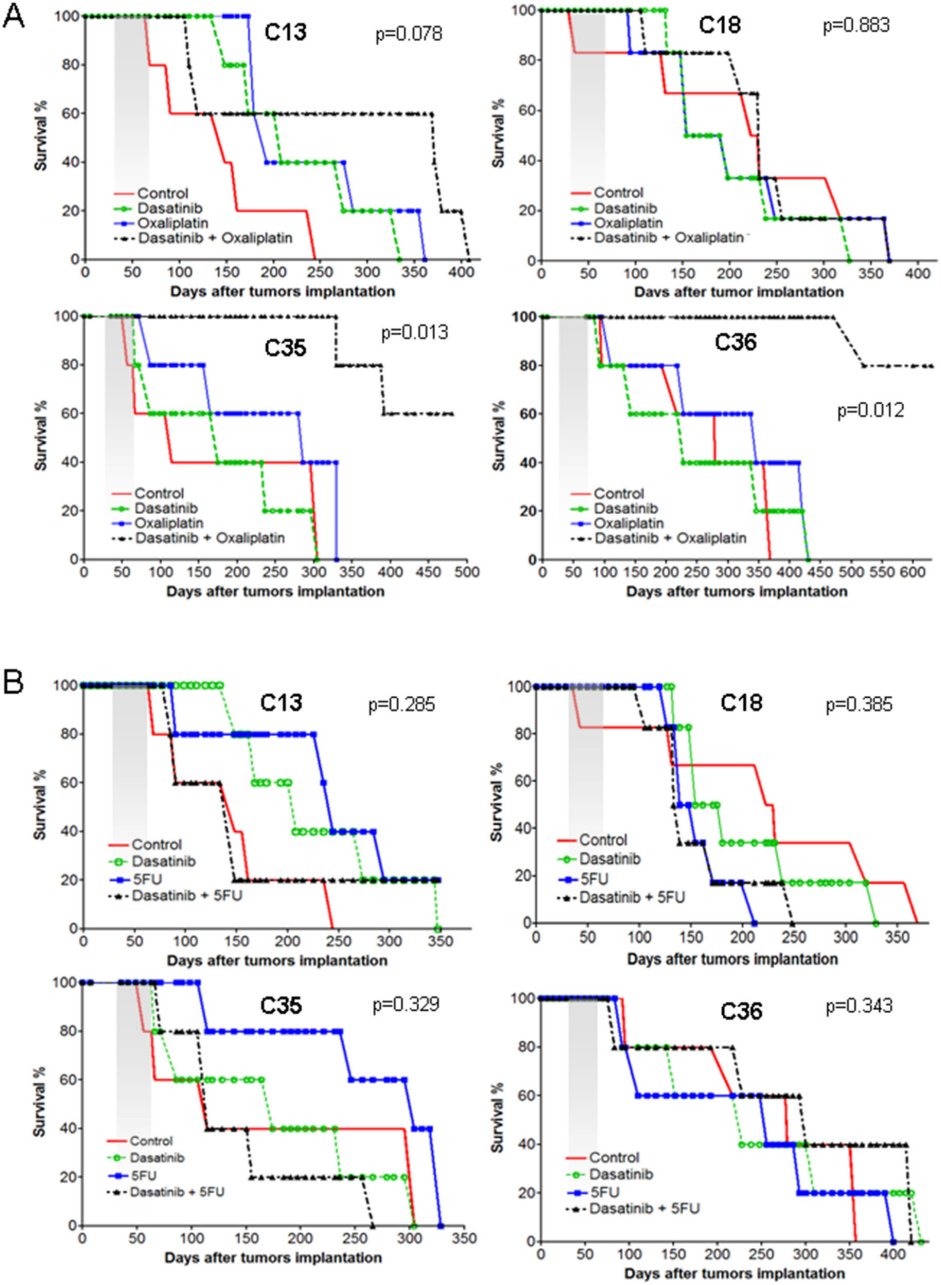
Survival curve of treated murine cohorts The four liver metastases of CRC tumor-derived PDXs were orthotopically engrafted into murine liver and grown for 15 days to ensure proper engraftment. Mice were allocated ad random to conform the cohorts and treated for 4 weeks as indicated in M&M. Then, the mice were maintained and observed daily. At signs of distress or illness the mice were sacrificed. **A.** Cohorts of mice treated with solvent alone, oxaliplatin, dasatinib or a combination of dasatinib+oxaliplatin. **B.** Cohorts of mice treated with solvent alone, 5FU, dasatinib or a combination of dasatinib+5FU.

To explore the effects of different treatments on these tumors, we treated for 4 weeks with oxaliplatin, the p-Src inhibitor dasatinib, a combination of both drugs (oxaliplatin plus dasatinib) or solvent (untreated) and then analyzed the survival of these mice (Figure 5A). We observed that the survival curves of mice carrying PDX tumors were not significantly different between untreated mice and mice treated with oxaliplatin or dasatinib alone. These results were independent of the levels of p-Src in each model. However, we observed clear and statistically significant improvement in the survival of mice with high p-Src that were treated with a combination of oxaliplatin plus dasatinib (Figure 5A). This improved survival was not observed in mice with low p-Src expression.

**Figure 6:**
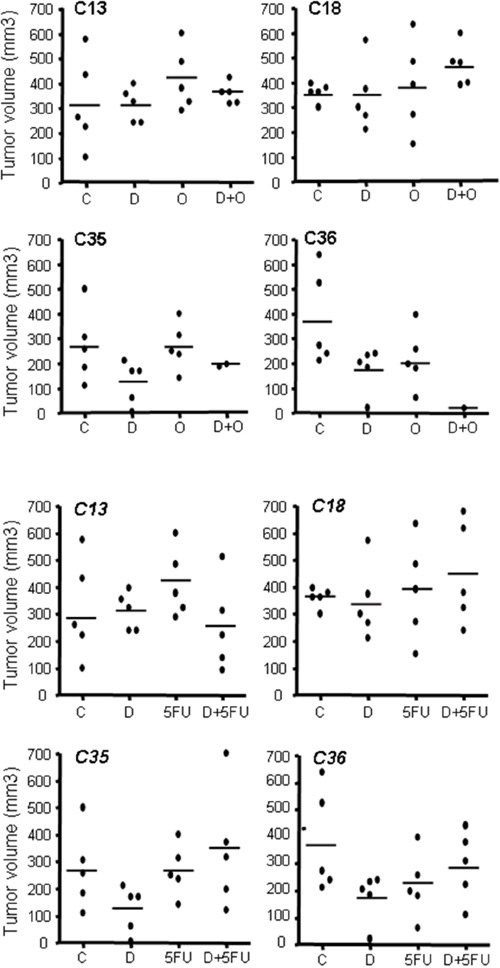
Size of PDX tumors in all cohorts at endpoint After sacrifice by humane end-point, all mice were necropsied and the tumor growing in liver measured. Each point represents the volume of the tumor found in each mice treated in our experiments.

The analysis of tumor size at various end points indicated a similar, albeit heterogeneous, size for all tumors (Figure 6), suggesting that survival was determined by tumor growth.

Of note, the increased efficacy of dasatinib plus oxaliplatin, as compared to either drug alone, observed in mice with CRC liver metastases was specific for oxaliplatin. We repeated the experiment with 5FU, another drug that is commonly used for late stage CRC tumors [31], but no clear improvement in survival was detected in mice that were treated with 5FU alone or in combination with dasatinib (Figure 5B).

*In vivo* p-Src inhibition by dasatinib is sinergistic with oxaliplatin in treating CRC liver metastasis, suggesting that p-Src is a possible biomarker for selecting patients who may benefit from this combination therapy.

## DISCUSSION

Despite advances in the development of new chemotherapeutic agents, advanced colorectal cancers remains an essentially incurable disease, as all patients eventually develop drug resistance, resulting in disease progression and death. Thus, therapeutic strategies that re-sensitize tumors to these agents may improve outcomes [32]. In this work, we demonstrated that one mediator of oxaliplatin sensitivity/resistance in some colon tumor cells is the active tyrosine kinase, Src. The cytoprotective effect of p-Src is specific to oxaliplatin and does not affect the tumor cell response to 5FU. Furthermore, p-Src (phosphorylated at Tyr419) is a good marker for predicting the activity of dasatinib in restoring oxaliplatin sensitivity *in vivo*.

Src activation promotes tumor progression and is associated with an aggressive phenotype and a poor prognosis in CRC [32]. C-Src kinase activity is balanced by the orchestrated function of protein kinases and phosphatases that mainly target Tyr530 at the C-terminal tail. C-terminal Src kinase (Csk) operates as a negative regulator, phosphorylating c-Src at the C-terminal segment [33]. Additionally, several cytoplasmic and transmembrane protein tyrosine phosphatases (PTPs) have been found to dephosphorylate c-Src at Tyr530, regulating its kinase activity [34]. Downregulation of the transmembrane PTPa has been proven to inhibit Src kinase activity and induce apoptosis in CRC cells [35]. Moreover, splice mutants of receptor-like PTPa (RPTPa) have been detected in 30% of colon, breast and liver tumors. Specifically, the expression of the RPTPa245 mutant in tumors facilitates the activation of Src via RPTPa binding [36]. In conjunction with the genetic regulation of Src, further epigenetic regulation of Src has emerged as an additional factor in CRC tumorigenesis [32, 37], including micro-RNAs. C-Src upregulation was correlated with miR-542-3p downregulation. Upregulation of integrin-linked kinase (ILK) follows, which promotes further c-Src and focal adhesion kinase (FAK) activation, as well as tumorigenic and invasive potential [38, 39]. Whether Src mediates growth factor receptor signaling leading to mitogenesis of CRC cells has not been clearly demonstrated. Contradictory results show that an elevated c-Src level does not directly influence proliferation neither *in vitro* or *in vivo* in CRC, but it is now accepted that c-Src-dependent cell cycle regulation is integrated through multiple interactions with membrane receptors and their downstream mitogenic signaling pathways [14, 16, 27, 40, 41]. It has been described that c-Src also enhances resistance to apoptosis by accelerating the ubiquination and proteasomal degradation of the protein Bcl-2 interacting killer (Bik) via the MAP kinase signaling pathway in CRC and lung cancer cells [16, 25, 40].

Dasatinib is a dual oral inhibitor of Src/V-abl Abelson murine leukemia viral oncogene homolog (Abl), which also targets a number of other Src family kinase members and receptor tyrosine kinases (RTKs), such as c-kit, PDGFRa, PDGFRb, Ephrin receptors and discoidin domain receptor 1 (DDR1). Dasatinib is already approved for the treatment of chronic myeloid leukemia and Philadelphia chromosomepositive acute lymphoblastic leukemia [42]. In CRC cell lines, dasatinib inhibits integrin-dependent cell adhesion and migration, with parallel inhibition of c-Src activity. Moreover, dasatinib has been found to inhibit FAK and paxillin activation [42–44]. However, the most relevant finding is the *in vivo* detection of a correlation between the inhibition of c-Src activity in peripheral blood mononuclear cells and the inhibition of kinase activity in tumors. This observation suggests peripheral blood may provide a useful surrogate tissue for biomarker studies with dasatinib [45].

To date, Src inhibitors as monotherapy have not yielded promising results [46, 47]. However, the use of Src inhibitors in combination with cytotoxic chemotherapy or RTK inhibitors seems more encouraging. Several early clinical trials are currently assessing these combinations in advanced solid tumors and metastatic CRC and shall provide further insights of this therapeutic approach in the near future [20, 32].

Combination therapy with dasatinib and oxaliplatin in a CRC metastatic murine model resulted in smaller tumors than therapy with either agent alone. Oxaliplatin was proven to activate c-Src through a ROS-dependent mechanism, and dasatinib-mediated c-Src inhibition sensitized cells to oxaliplatin activity [48]. Our *in vitro* and *in vivo* data showed that Src inhibition is effective only in CRC liver metastasis with high p-Src expression at Tyr419, which also correlates with an increased activation of MAPKs. In metastatic CRC, Kras mutations are predictive biomarkers of resistance to cetuximab. However, dasatinib has been found to more effectively treat Kras-mutant CRC cell lines than cetuximab, and the administration of both drugs shows better anti-proliferative effects *in vitro* and *in vivo* [49]. Consistent with this, the presence of Kras mutations in our models is irrelevant to dasatinib-mediated chemosensitization to oxaliplatin *in vitro* and *in vivo*. Reduced SFK activity by molecular and pharmacological inhibition also decreases VEGF expression *in vitro* and seems to suppress neo-vascularization *in vivo* [50][51][52]. Based on these observations, it has been postulated that dasatinib may prevent increases in plasma VEGF during treatment with the VEGF-targeted monoclonal antibody bevacizumab, and may thereby enhance the effect of anti-angiogenic therapies. Dasatinib is in fact currently being tested in combination with capecitabine and bevacizumab in previously untreated metastatic CRC patients.

At this point we do not know the molecular mechanism that precludes dasatinib sensitizing these tumors to 5FU. It is possible that a higher level of ROS induced by oxaliplatin may influence the response by additional activation of Src [48]. This additional activation will make these cells yet more dependent on Src activity for survival through an oncogenic addiction-like mechanism.

In summary, dasatinib effectively sensitizes CRC liver metastasis to oxaliplatin in orthotopically-grown patient-derived xenografts, However, dasatinib has this effect only in tumors with high Src phosphorylation at Tyr419. As a result, high Src phosphorylation is a good prognostic and predictive marker that can be easily tested in ongoing clinical trials.

## MATERIALS AND METHODS

### Human colorectal adenocarcinoma cell lines, culture conditions and transfection

COLO-205, SW48, SW480, LS180, LS174-T, HT-29, T84 and LoVo cells were purchased from the European Collection of Cell Cultures (ECACC). Cells in culture were maintained as a subconfluent monolayer in Dulbecco's Modified Eagle's medium supplied with non-essential amino acids (LS180 and LS174-T), Dulbecco's modified Eagle's medium-F12 nutrient mixture (T84), McCoy 5-A medium (HT-29), L15 Leivobitz medium (SW48 and SW480), Nutrient Mixture F12 HAM (LoVo) and RPMI 1640 (Colo-205) purchased from Sigma. Each cell line was grown under identical conditions, and cell culture medium supplements were provided according to the manufacturer's instructions. To ectopically overexpress c-Src kinase protein, subconfluent SW480 and T84 cells were transfected with 0.4 μg of pBABE-puro expression plasmid carrying cDNA from a *c-Src* gene. Stable clones of the T84 cell line were selected in F12/DMEM medium supplied with 0.5 μg of puromycin for 3 weeks. In the same way, stable clones of the SW480 cell line were selected in L15 Leivobitz medium supplied with 0.5 μg of puromycin. As a control, subconfluent SW480 and T84 cells were transfected with 0.4 μg of DNA containing an empty pBABE-puro expression plasmid. Positive clones were selected for protein expression measured by Western blotting. The entire set of transfected clones was used as a stable pool for transfection.

### Western blotting

Cells were washed twice in PBS, lysed in RIPA lysis buffer (Tris–HCl pH 8.0 25 mM, NaCl 150 mM, NP40 1%, sodium deoxycholate 1%, SDS 1%, Na3VO4 1 mM, EDTA 0.5 M, and complete protease and phosphatase inhibitor cocktail 2 mM) and subjected to a 3x sonication burst cycle for 5 seconds at 30-40%. The cells were ultimately pelleted for 30 seconds at 14000× g. After centrifugation, supernatant protein extracts were aliquoted and stored at −80°C until use. Protein levels were determined by Bradford assay using BSA (bovine serum albumin) as a standard. The appropriate protein quantity was dissolved in Laemli buffer (Tris–HCl, pH 6.8, 62.5 mM, glycerol 10%, SDS 1%, 2-mercapto ethanol 5%, bromphenol blue 0.0025%), and the proteins were separated on SDS-PAGE gels (12%) before they were blotted onto a nitrocellulose transfer membrane (Whatman - Protrans). The primary antibodies included the following: anti-Src antibody 1:2500 [EG107] – (Abcam ab32102), anti-Src (phospho Y419) antibody – (Abcam ab4816), p44/p42 MAPK (Erk1/2) Rabbit mAb 1:1000 (Cell Signaling 137F5), and tubulin 1:10000 (Sigma – T6557). The secondary antibodies included goat anti-rabbit Alexa Fluor 680 1:5000 (Invitrogen – A21057) and donkey anti-mouse IRDye 800CW 1:5000 (Rockland Inc. – 605-731-002). To inhibit c-Src, an inhibitor of Src family kinases, PP2 (Sigma #P0042-5MG), was supplied at a 10 μM concentration overnight before protein extraction.

### Cytotoxicity assay

Oxaliplatin, 5-Fluorouracil (5FU) and PP2 (a Src inhibitor) were freshly and individually prepared in deionized water (Oxaliplatin 10 mM, 5FU 32.09 mM) or DMSO (PP2 30 mM) for each experiment. Cell lines were seeded in 96-well plates (2.000-3.000 cells per well depending on the cell size). The treatment was assayed after the application of decreasing concentrations in a 1:3 fixed ratio to exponential phase growing cells. All treatments were applied within decreasing doses of Oxaliplatin, 5FU and PP2 individually or in a fixed ratio 24 hours after seeding. Proliferation was determined by MTT assay after 96 hours. Cytotoxicity was measured by absorbance at 595 nm using a microplate reader (BIORAD iMark™ Microplate Reader); then, the IC50 was estimated using GraphPad Prism 4 software. For combined treatments, PP2 (10 μM) were plated with decreasing doses of both Oxaliplatin and 5FU at a fixed ratio. Combination indices were obtained by previous suboptimal doses of Oxaliplatin, 5FU and PP2 alone.

### Human metastatic tumors samples

Tumor samples were collected from patients undergoing surgical resection of CRC liver metastasis at Hospital Universitario Virgen del Rocío (Seville, Spain). All patients previously provided a signed consent according to a study protocol approved by the local ethics committee (CEI 2012/PI007).

### Patient derived xenograft (PDX) generation

Tumor samples derived from colorectal cancer liver metastases were resected and freshly collected from patients. Then, they were maintained in Dulbecco's modified Eagle's medium nutrient mixture/F10 (DMEM/F10 Sigma) containing 10% fetal bovine serum, penicillin, streptomycin and amphotericin B (100 mg/ml each; Sigma). The samples were maintained for less than 2 hours in cell culture medium at room temperature before orthotopic implantation. Each tissue was divided into 2 fragments. One fragment was frozen for further molecular analysis, and the remaining tumor sample was divided into fragments that ranged from 2-3 mm and were eventually used for orthotopic liver implantation in 6-week-old Foxn1nu athymic nude female mice (Harlan Laboratories, Netherlands). The mice were anesthetized with ketamine. After laparotomy, an area of 3×3 mm was dissected in the anterior side of the left hepatic lobe. Inside the dissected cavity human metastasis of CRC was implanted. Neoplastic tissue grew inside liver tissue. Six months later, all mice were euthanized, and the tumors were individually re-implanted and grown in a similar setting, allowing experiments within a physiologically and molecularly similar setting. These experiments were performed according to the European guidelines for laboratory animal care. This study was approved by the IBIS Institutional Animal Care and Use Committee.

### *In vivo* treatments

Each tumor sample was xenografted individually into nude mice. After sufficient tumor growth, the mice were euthanized, and their tumors were collected and divided onto 2×2×2 mm blocks and then orthotopically re-implanted in mice liver. Experiments were performed using 6-murine cohorts. Mice were randomly allotted to drug-treatment using Oxaliplatin, 5FU, dasatinib, solvent and combined treatments. Five weeks after tumor implantation, all mice were treated for 4 weeks (2-5 times per week depending on the treatment type). Each symptom of murine distress was periodically verified twice a week and the murine weight was measured at the same time. *In vivo* experiments were completed at a clinical endpoint according to the IBIS Institutional Animal Care and Use Committee. Oxaliplatin and 5FU were obtained from the pharmacy at Hospital Universitario Virgen del Rocío, freshly prepared and intraperitoneally administered at 1 mg/Kg/dose over a period of 4 weeks (twice a week, different days). Dasatinib, (BMS-354825) was freshly prepared and administered at 20 mg/Kg/dose over a period of 4 weeks (5 times per week, on different days).

### Necropsy procedures

The experiments were terminated when the animals reached a human equivalent end-point. All mice were eventually sacrificed and necropsied. Tumors were excised and measured using a caliper according to the following equation: tumor volume = [length x width^2^]/2.

## SUPPLEMENTARY FIGURES AND TABLES


