# Correction: Dasatinib, a Src inhibitor, sensitizes liver metastatic colorectal carcinoma to oxaliplatin in tumors with high levels of phospho-Src

**DOI:** 10.18632/oncotarget.13482

**Published:** 2016-11-21

**Authors:** Marco Perez, Antonio Lucena-Cacace, Luis Miguel Marín-Gómez, Javier Padillo-Ruiz, Maria Jose Robles-Frias, Carmen Saez, Rocio Garcia-Carbonero, Amancio Carnero

Present: Due to an error in proofreading, a research project number was accidentally omitted from the Acknowledgments list.

Correct: The correct numbers are provided below. The authors sincerely apologize for this oversight.

Original article: Oncotarget. 2016; 7(22):33111-24. doi: 10.18632/oncotarget.8880.

