# Supplementary material for: Dasatinib, a Src inhibitor, sensitizes liver metastatic colorectal carcinoma to oxaliplatin in tumors with high levels of phospho-Src
Source: Oncotarget. 2016 Apr 20;7(22):33111–24. doi: 10.18632/oncotarget.8880 (PMC5078079; doi:10.18632/oncotarget.8880)
Supplement: Supplementary file 1 [file oncotarget-07-33111-s001.pdf]

Dasatinib, a Src inhibitor, sensitizes liver metastatic colorectal carcinoma to oxaliplatin in tumors with high levels of phospho-Src

SUPPLEMENTARY FIGURE AND TABLE

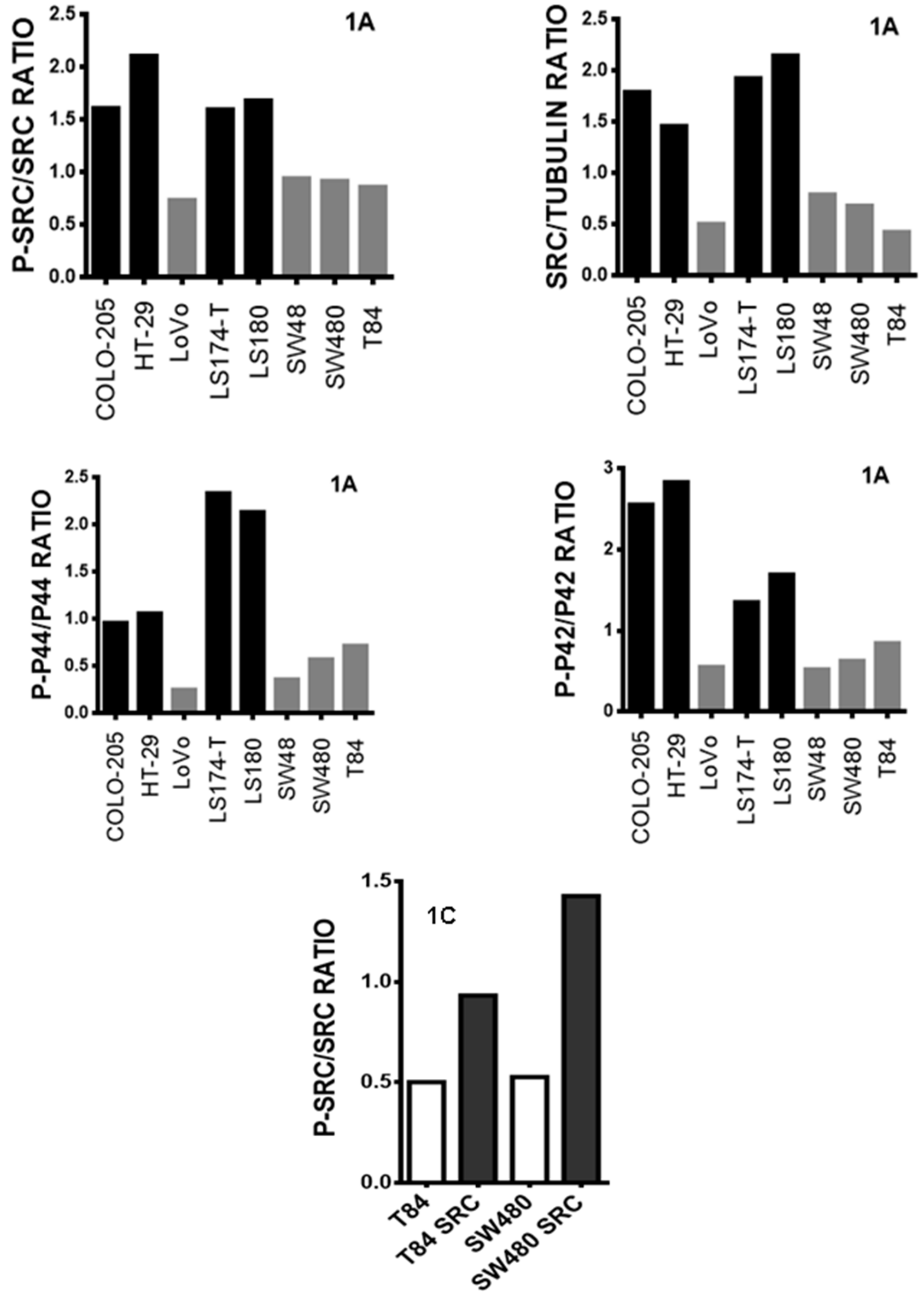

Supplementary Figure S1: Quantification of the protein levels of figure 1.

Supplementary Table S1: Mutational analysis of the CRC lines used in this study

Cell line	Kras Mutation	Braf V600E	PIK3CA Mutation
COLO 205	Wild type	Mutated	G914R
HT29	Wild type	Mutated	P449T
LS174T	G12D	Wild type	H1047R
LS180	G12D	Wild type	H1047R
LOVO	G13D	Wild type	
T84	G13D	Wild type	E542K; H1047R
SW480	G12V	Wild type	
SW48	Wild type	Wild type	G914R

The mutational status was obtained from COSMID database and confirmed by our own mutational analysis.
